# Supplementary material for: Machine learning prediction of antiviral-HPV protein interactions for anti-HPV pharmacotherapy
Source: Sci Rep. 2021 Dec 21;11:24367. doi: 10.1038/s41598-021-03000-9 (PMC8692573; doi:10.1038/s41598-021-03000-9)
Supplement: Supplementary file 4 — Supplementary Table S4. [file 41598_2021_3000_MOESM4_ESM.docx]

**Supplementary Table 4**. Metrics of KNN and SVM used for predicting antiviral-HPV protein interaction.

| **Predictor** | **Precision** | **Recall** | **F1-measure** | **Accuracy** | **AUC** |
| --- | --- | --- | --- | --- | --- |
| SVM | 0.80 | 0.23 | 0.36 | 0.59 | 0.59 |
| KNN | 0.85 | 0.64 | 0.73 | 0.76 | 0.76 |
